# IgG4-Related Ophthalmic Disease Presenting as Meningitis and Panuveitis

**DOI:** 10.1155/2019/5653282

**Published:** 2019-07-15

**Authors:** Maria A. Mavrommatis, Sarah A. Avila, Richard France

**Affiliations:** ^1^Department of Ophthalmology, Icahn School of Medicine at Mount Sinai, 1 Gustave L. Levy Pl, New York, NY 10029, USA; ^2^James J Peters VA Medical Center, Department of Ophthalmology, 130 W Kingsbridge Rd, Bronx, NY 10468, USA

## Abstract

**Purpose:**

We report an uncommon case of immunoglobulin gamma 4-related ophthalmic disease (IgG4-ROD) presenting as meningitis and panuveitis.

**Observations:**

A 35-year-old male with no prior ophthalmic history presented with headaches, altered mental status, and fever of unknown origin. A lumbar puncture (LP) revealed an elevated white count with lymphocytic predominance, confirming a suspected meningitis. After an extensive work-up, he was discharged on oral acyclovir to cover for presumed aseptic meningitis. The patient initially improved, however, bilateral eye pain, redness, and photophobia 2 weeks after discharge prompted his first visit to the ophthalmology clinic. Exam at that time was consistent with bilateral anterior uveitis for which he was given topical prednisolone and cyclopentolate. In addition to the preceding work-up, quantitative immunoglobulin serology including IgG4 levels was added. At follow-up, he was found to have increased ocular inflammation with vitreitis, nerve head edema, and subclinical macular thickening. Visual acuity (VA) had decreased in both eyes. Serology titers for IgG had resulted in a significant elevation in IgG subclass 4 (IgG4). Optical coherence tomography (OCT) and fundus fluorescein angiography (FFA) confirmed posterior retinal involvement. The patient was diagnosed with presumed bilateral panuveitis secondary to IgG4-ROD.

**Conclusions and Importance:**

IgG4-RD can be a serious condition that requires careful consideration and intuition to diagnose. This report serves to encourage ophthalmologists to consider IgG4-ROD in cases of idiopathic systemic inflammation with ophthalmic involvement.

## 1. Introduction

Immunoglobulin G4-related disease (IgG4-RD) is a recently discovered inflammatory condition characterized by IgG4-mediated fibroinflammation and enlargement of the affected organ [1]. While the notion of IgG4-RD was first recognized in the context of autoimmune pancreatitis [2], the disease has since been connected to inflammatory processes in many other organs, including the orbit and the eye [3]. IgG4-related ophthalmic disease (IgG4-ROD) is the term coined for instances of IgG4-RD resulting in inflammation of the ocular adnexa. IgG4-ROD has multiple clinical presentations, most frequently involving the lacrimal gland, extraocular muscles, orbital bones, and sclera [4]. Few case reports document meningeal involvement and even fewer present with alternative ocular pathology. We report an atypical case of IgG4-ROD presenting as aseptic meningitis and panuveitis.

## 2. Case Report

**Figure 1:**
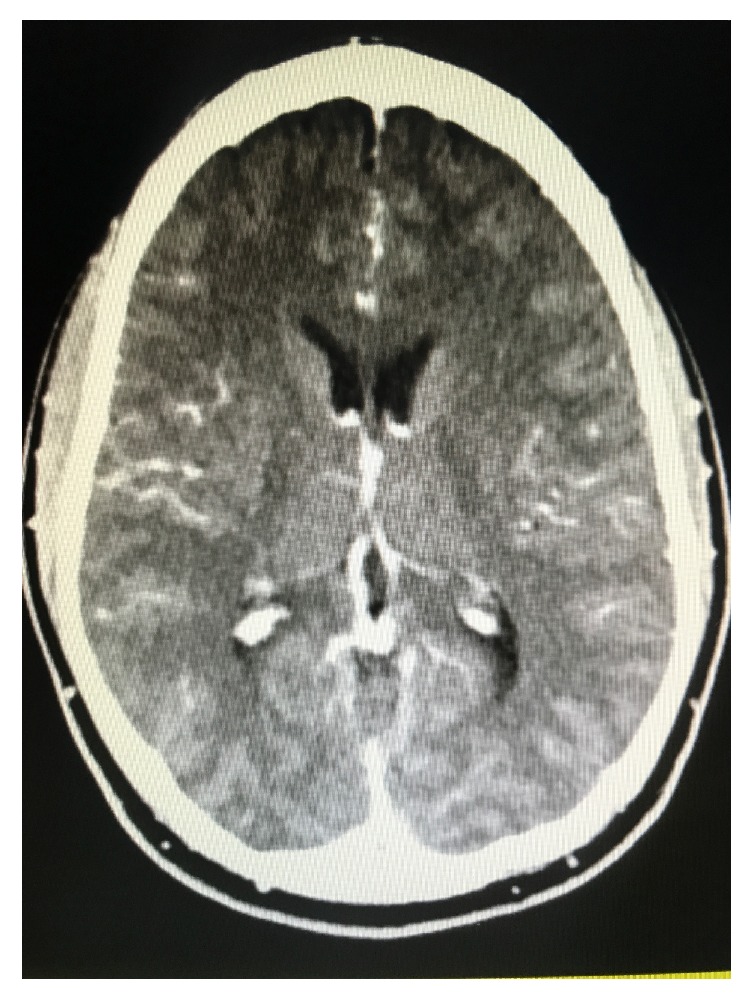
CT Scan showing diffuse inflammation and thickening of the meninges.

A 35-year-old male with no prior medical or ophthalmic history presented with headaches, altered mental status, and fever of unknown origin. An extensive work-up for infectious, inflammatory, and neoplastic causes was noncontributory. Labs revealed marked elevations in both erythrocyte sedimentation rate (ESR) and C-reactive protein (CRP), indicating an underlying inflammatory process. Computer tomography (CT) scan of the head showed diffuse inflammation and thickening of the meninges (Figure 1). Lumbar puncture (LP) revealed an elevated white count with lymphocytic predominance. He was discharged on a course of oral acyclovir for presumed aseptic meningitis.

**Figure 2:**
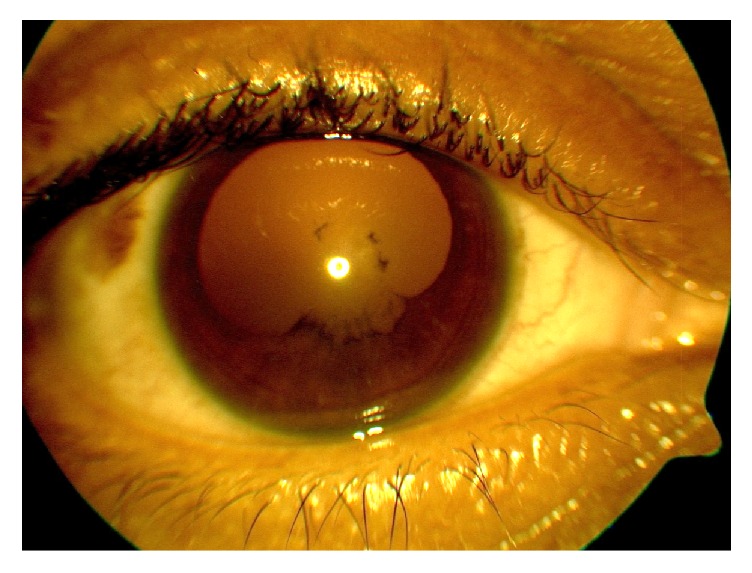
External photo of the left eye following synechiolysis with pigmentary ring on the anterior lens capsule.

**Figure 3:**
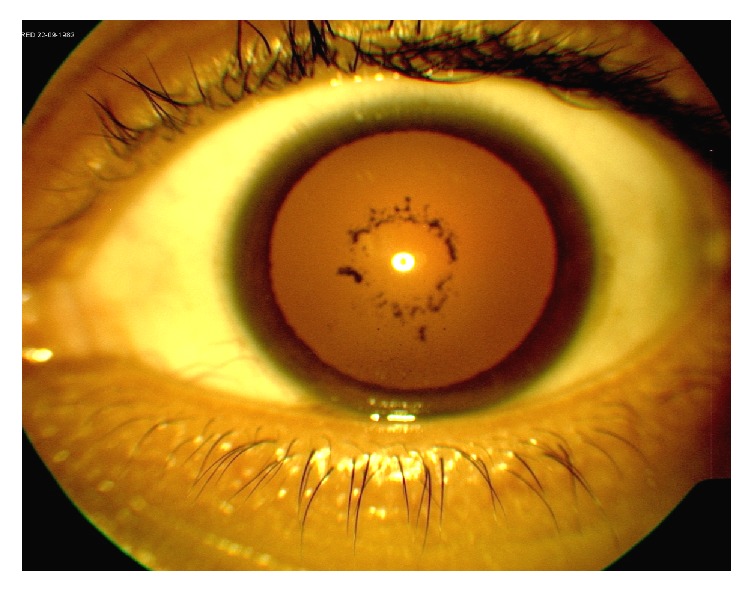
External photo of the right eye demonstrating posterior synechiae with pigment on the anterior lens capsule.

The patient initially improved; however, two weeks after discharge, bilateral eye pain, redness, and photophobia prompted his first visit to the ophthalmology clinic. Slit lamp exam was notable for grade 2 anterior chamber cells with flare in both eyes. The right eye showed posterior synechiae with pigment on the anterior lens capsule (Figure 2) while the left eye showed synechiolysis with a residual pigmentary ring on the anterior lens capsule (Figure 3). Dilated fundus exam was within normal limits. He was given topical prednisolone and cyclopentolate for a diagnosis of bilateral anterior uveitis. In addition to his preexisting work-up, quantitative immunoglobulin serology including IgG4 levels was also added given the unexplained systemic inflammation.

**Figure 4:**
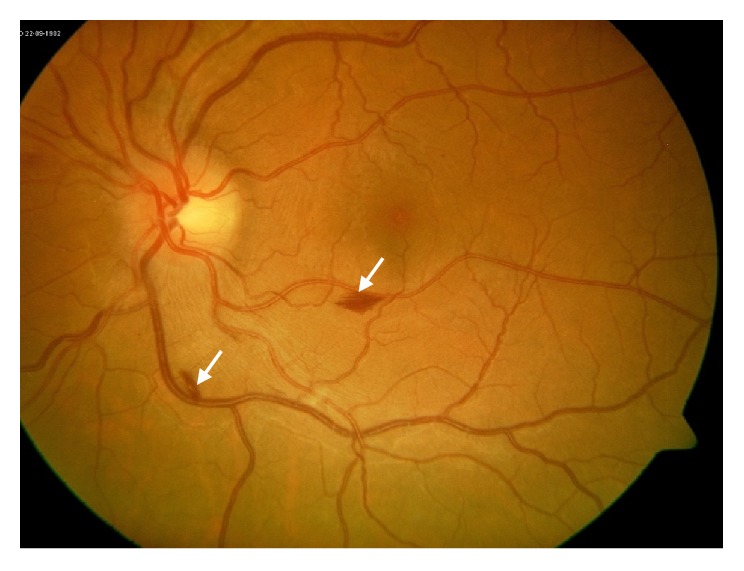
Fundus photo of the left eye demonstrating optic nerve head edema with flame hemorrhages along the inferior vascular arcade (white arrows).

**Figure 5:**
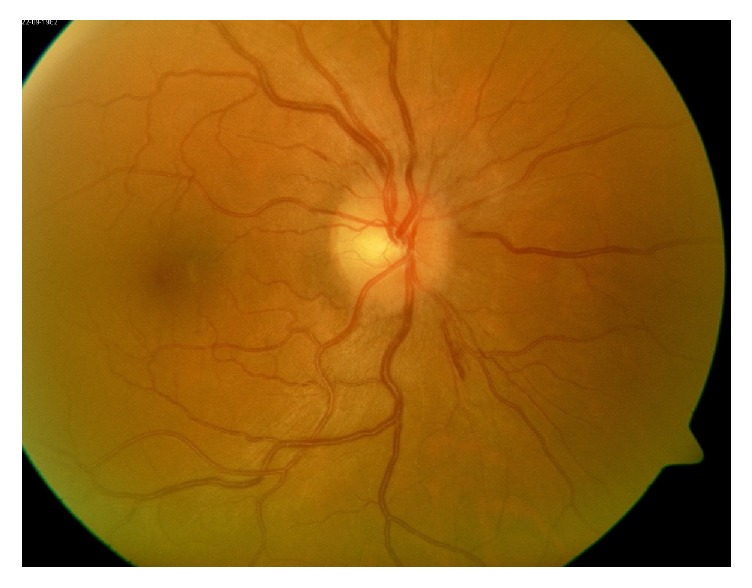
Fundus photo of the right eye demonstrating optic nerve head edema.

**Figure 6:**
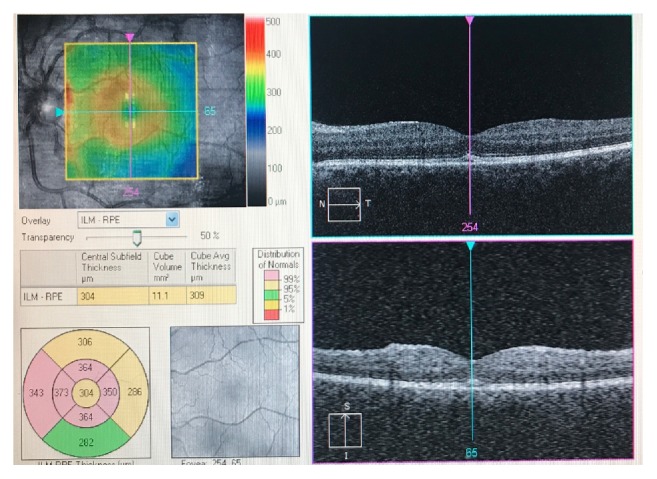
OCT Macula of the left eye demonstrating para-foveal macular thickening.

**Figure 7:**
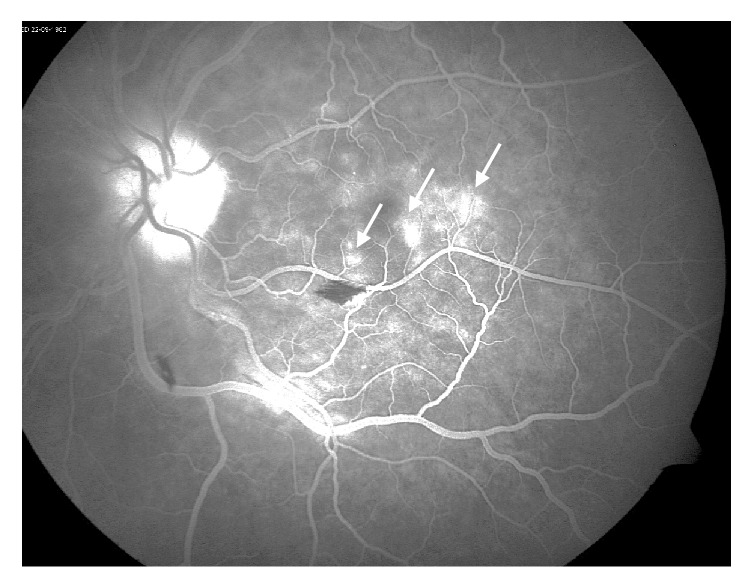
Fundus fluorescein angiography of the left eye showing para-foveal late vascular leakage (white arrows) and blockage from flame-hemorrhages.

**Figure 8:**
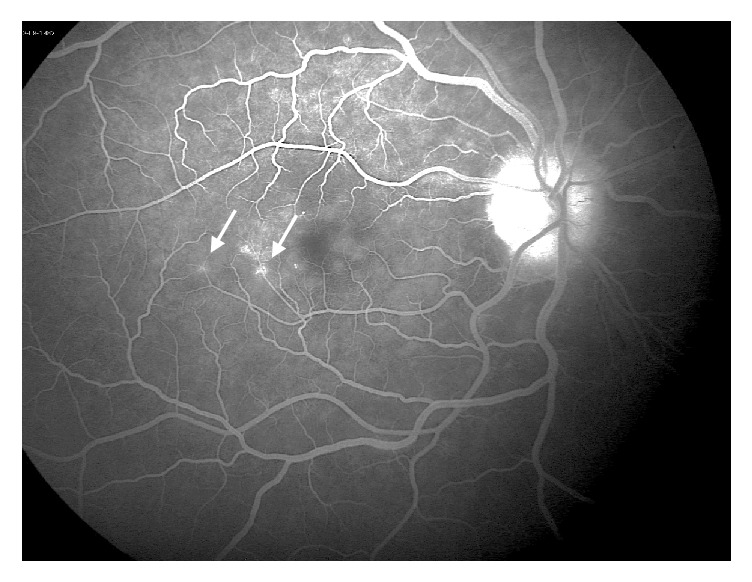
Fundus fluorescein angiography of the right eye showing para-foveal late vascular leakage (white arrows).

At follow-up, his symptoms persisted with a decrease in vision from 20/20 in both eyes to 20/25. He was found to have increased ocular inflammation with persistent anterior chamber cells and subsequent vitreitis and bilateral optic nerve head edema in both eyes (Figures 4 and 5). Optical coherence tomography (OCT) was normal in the right eye but showed subclinical parafoveal thickening in the left eye (Figure 6). Fundus fluorescein angiography (FFA) confirmed posterior retinal involvement with bilateral parafoveal late vascular staining (Figures 7 and 8).

**Figure 9:**
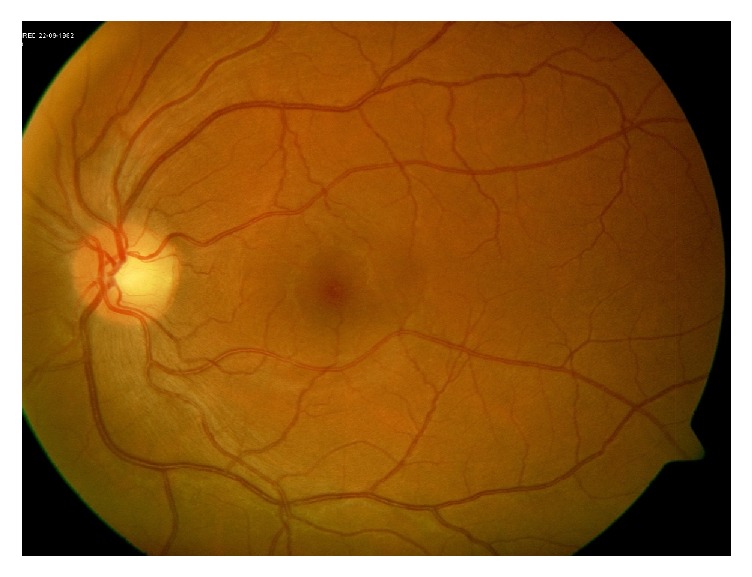
Fundus photo of the left eye after treatment showing resolution of the optic nerve head edema.

**Figure 10:**
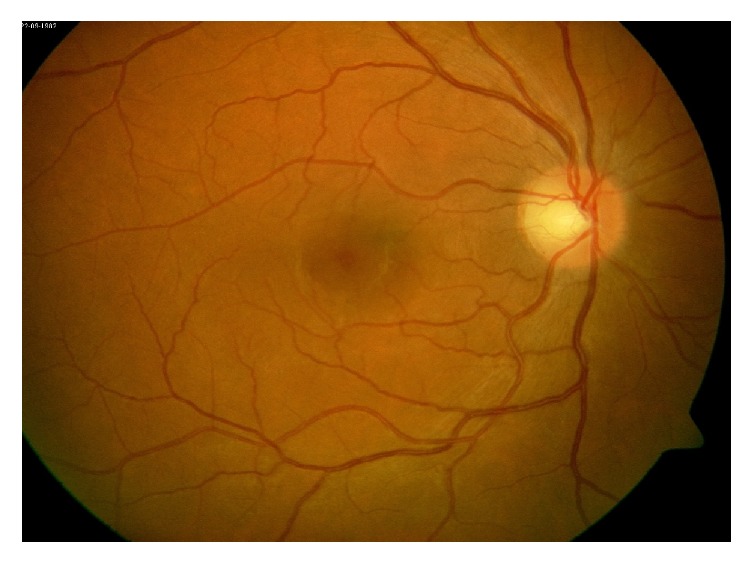
Fundus photo of the right eye after treatment showing resolution of the optic nerve head edema.

**Figure 11:**
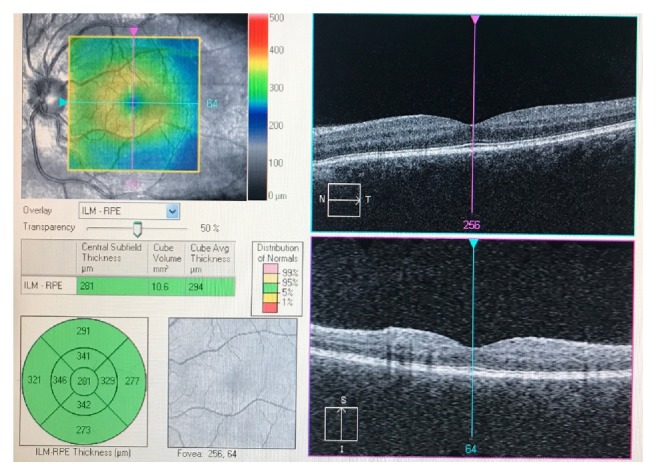
OCT Macula of the left eye after treatment demonstrating improved para-foveal macular thickening.

Serology titers for IgG revealed a significant elevation in IgG subclass 4 (IgG4) at 251 mg/dL. A presumed diagnosis of bilateral panuveitis secondary to IgG4-ROD was made. The patient was offered a vitreous biopsy to further confirm the diagnosis, but he declined. He was treated with high dose oral prednisone at 60 mg/day with full resolution in anterior chamber cells, optic nerve head edema (Figures 9 and 10), and OCT macular swelling (Figure 11). The patient's visual acuity returned to 20/20 after treatment.

## 3. Discussion and Conclusion

IgG4-RD is an increasingly recognized immune-mediated condition that can yield destructive inflammation and fibrosis in a number of organs [1]. When the disease manifests in the eye as IgG4-ROD, it typically presents with lacrimal gland, extraocular muscle, and, less commonly, eyelid, orbital bone, or scleral involvement [3]. These manifestations can mimic many other conditions, including neoplastic, infectious, and inflammatory diseases. Thus, these require exclusion before a final diagnosis of IgG4-RD can be entertained. Diagnostic criteria put forth by Umehara et al. for IgG4-RD and by Goto et al. for IgG4-ROD can help characterize definitive, probable, and possible IgG4 disease [5, 6]. Furthermore, Yu et al. determined that the optimal cutoff value of IgG4 serum for the diagnosis of IgG4-RD is 248 mg/dL, with a sensitivity and specificity of 77.6% and 92.8%, respectively [7]. They subsequently concluded that 2 or 3 times the upper limit of the normal range of IgG4 level, as was found in the present case, is a useful marker for the diagnosis of IgG4-RD [7]. While serum IgG4 elevated levels significantly aid in the diagnosis, the gold standard for a definitive diagnosis is tissue biopsy. Using serological cut-offs like those put forth by Yu et al. is advantageous in situations when biopsy is difficult or when the patient declines, as in this case.

Here, we describe a rare case of probable IgG4-ROD presenting as meningitis and progressive panuveitis that required an extensive work-up to identify. While IgG4-RD is typically diagnosed by rheumatological specialties, we advise ophthalmologists to consider IgG4-ROD and to add IgG serology to the work-up in cases of idiopathic inflammation. When the presentation varies beyond the expected, the risk of misdiagnosing the condition heightens, which can be detrimental to the patient's capacity for recovery. A condition like IgG4-ROD can offer an opportunity for elevated collaboration between ophthalmology and rheumatology for a final diagnosis. Despite favorable responses to steroids, long-term management of relapsing IgG4-RD and igG4-ROD patients can be challenging, further supporting the need for awareness and multidisciplinary cooperation as it may require immunomodulatory therapy.
